# On the Use of the Extract of Belladonna, and Buchu Leaves, in Spasmodic Strictures of the Urethra

**Published:** 1831-04

**Authors:** Edwin Long


THE LONDON

### Medical and Physical Journal.

No. 386, VOL. LXVI.] APRIL 1831. [No. 58, *New Series.*

**"For many fortunate discoveries in medicine, and for the detection of numerous errors, the world is indebted to the rapid circulation of Monthly Journals; and there never existed any work to which the Faculty, in Europe and America, were under deeper obligations than to the 'Medical and Physical Journal of London,' now forming a long but invaluable series."—RUSH.**

### ORIGINAL PAPERS, AND CASES,

### OBTAINED FROM PUBLIC INSTITUTIONS AND OTHER AUTHENTIC SOURCES.

### SPASMODIC STRICTURES OF THE URETHRA.

*On the Use of the Extract of Belladonna, and Buchu Leaves, in Spasmodic Strictures of the Urethra.* By EDWIN LONG, Esq.

***To the Editor of the London Medical and Physical Journal.***

SIR: On reading your review of Mr. MACILWAIN's Treatise on Strictures of the Urethra, I was rather surprised to find that no mention was made of the use of the Ext. Belladonnæ in spasmodic stricture of that part, as well as in the irritable urethra; having an impression on my mind that I have somewhere seen it recommended, although I fancy that I was the first person who ever employed it in that way, and this I have been in the habit of doing ever since the year 1820.*

Now, sir, I consider it the imperative duty of every member of the profession, if he has discovered any thing likely to

* Our Journal for November 1826, p. 403, contains a paper, by Mr. CHEVALIER, "on the External Use of the Extract of Belladonna." Among other interesting remarks offered by Mr. C., a case of stricture in the urethra is related, in which he introduced a bougie anointed with belladonna extract, for the purpose of relieving the excessive pain the patient had previously experienced, when a common bougie was passed. The practice was useful; for, "after the bougie, thus prepared, had pressed upon the irritated part for about two minutes, the intense pain which the patient at first endured was mitigated, so much so, that larger bougies were successively introduced, until at length No. 14 was used without difficulty." Mr. Chevalier was not aware that this expedient had ever been had recourse to before. He also states, that he "had used a solution of Extr. Belladonnæ, combined with other contribute to the alleviation of the sufferings of his fellow-creatures, to communicate it freely, and without reserve. It may, perhaps, be asked, feeling thus, why I have so long delayed the disclosure? I answer, from a degree of diffidence, lest any thing which I might communicate should not be considered of sufficient importance to interest the profession. Yet, although I have made no public mention of the circumstance, I have fully disclosed it to many of my medical brethren.

The first case in which I employed it was in 1820, and I regret very much that I did not take notes at the time, but the leading particulars I can very well relate from memory. The subject was a man aged fifty; his occupation that of a waterman. I was sent for in the evening, and found him in very great pain, from an inability to void his urine: he told me, that he had been frequently under the care of respectable surgeons, some of whom he named, "for a *constitutional gravelly complaint;*" but that all of them failed in giving him more than temporary relief, the disease always returning upon exposure to cold, or too much exercise used in walking. He could, at that time, only pass his water by drops, and had only voided about a teacupful during the last twenty-four hours. I prescribed what I considered the most suitable medicines, and saw him again the following morning, but was sorry to find there was no alleviation of his sufferings: I therefore attempted to introduce the catheter, when I found a completely strictured urethra, the instrument not passing farther than about two inches along the canal. I then procured a series of bougies, and at length succeeded in passing a very small wax one as far as the membranous portion of the urethra, but beyond this it would not pass: suffice it to say, that, for three days after I saw him, every means were used which I had either seen recommended by others, or my own ingenuity would suggest, without effect; such as bleeding to syncope, hot bath, an attempt at introduction whilst in the bath, &c., but all to no avail.

The patient's state now became alarming; the bladder was distended to an enormous size, and I dreaded every moment ingredients, and especially the vegetable astringents, in many other cases of irritable urethræ, and of morbid tenderness in the vagina, with similar success."

We have also recorded another case, in which the same treatment was employed, with great advantage, by Mr. Tyrrell, at St. Thomas's Hospital. This patient had retention of urine, from stricture. (London Med. and Phys. Journal, April 1830, p. 326.) It is *said* that strangulated hernia has been reduced by the external application of belladonna. (Ibid. June 1829, p. 558.) —Editor.

lest it should give way: I therefore determined, as a dernier resort, to puncture it through the rectum. This I was almost upon the point of doing, when it struck me that, perhaps, the belladonna, from its peculiar relaxing powers, might exert a beneficial influence: with, therefore, the least possible delay, some extract was sent for, and, after reducing a portion of it with water, it was well rubbed along the whole course of the urethra, and particularly in the perineum. I then took a small bougie, and made a groove along it, as recommended by Mr. Carpue, smeared the extremity, and to the extent of an inch, well with the undiluted extract, and, without much difficulty, passed it as far as the membranous portion of the urethra; when, after remaining a few seconds, I could distinctly feel something give way, and it slipt into the bladder. The water escaped gradually along the groove made in the bougie, to the extent of many pints: I will not, as I took no notes, at this distance of time, undertake to mention the precise quantity. I remember perfectly that it was some days before I could introduce the smallest-sized catheter, and never that, so violent was the spasm, without the use of the belladonna.

The cure occupied the space of a twelvemonth. I preferred the use, for a considerable time, of the silver catheter to the bougie, because I found the former instrument answer two purposes: it both dilated the urethra, and also allowed the urine to escape; for several times, when, for experiment sake, I used the bougie at the time when the patient could not readily evacuate the contents of the bladder, a spasm immediately came on, which prevented the flow of water. The latter part of the cure, for some months, was completed by means of the metallic bougies; always previously immersing them in warm water, to render them, as near as possible, of the temperature of the part. I was somewhat surprised, from the free use of the belladonna in that case, that it did not occasion a temporary incontinence of urine, from its probable effect upon the sphincter muscle of the bladder: such, however, was not the case; nor have I ever seen any bad effects result from its application. Suffice it to say, however, that I never have, nor should I think of using it, except in extreme cases.

I believe that, had I then known of the Buchu leaves, I should have found them a very valuable adjunct. I have since very frequently prescribed them, and with very great advantage, in stricture, and different affections of the urinary organs.

*Pontefract.*

